# Risperidone Abruption-Induced Tardive Dyskinesia in a Six-Year-Old Male Patient With Known Autism and Attention Deficit Hyperactivity Disorder: A Case Report

**DOI:** 10.7759/cureus.31492

**Published:** 2022-11-14

**Authors:** Abdullah H Alhamoud, Abdullah Yatimi, Saad A Towheri, Hassan A Sharahili, Abdu M Hawas

**Affiliations:** 1 Pediatric Medicine, King Fahad Central Hospital, Jazan, SAU; 2 College of Medicine, Jazan University, Jazan, SAU; 3 Pediatric Neurology, King Fahad Central Hospital, Jazan, SAU

**Keywords:** tardive dyskinesia, risperidone, adhd, autism, pediatric

## Abstract

As a serotonin-dopamine antagonist, risperidone is less likely than traditional antipsychotics to result in tardive dyskinesia (TD). There are not many reports of risperidone abruption-induced TD. Herein we report a new case of tardive dyskinesia induced by a sudden stop of risperidone during the treatment of an autistic patient with attention deficit hyperactivity disorder (ADHD) on risperidone. He was presented to the emergency department in King Fahd Central Hospital in Jazan, Saudi Arabia, with a history of abnormal movement in the form of unsteady gait, axial dystonia, twisting and spreading of fingers, shoulder shrugging, and protruding tongue associated with hypersalivation, with no other signs and symptoms. These symptoms started after two days of abruption. The laboratory and imaging results showed normal findings. Other causes that induced symptoms were ruled out. The diagnoses of tardive dyskinesia were presumed. Risperidone was not restarted, and clonazepam was started with a gradual increase of the dose from 0.2 mg/twice a day for five days to 0.2 mg/three times a day. The patient's symptoms improved, and he was discharged with a follow-up with a psychiatrist and neurologist. Risperidone and other atypical second-generation antipsychotics were used to treat autism spectrum disorders. TD is more likely to be triggered by the abrupt withdrawal of risperidone. The chosen laboratory tests and imaging tests are helpful in ruling out other causes that induce similar symptoms and presumed diagnosis of TD. The conventional recommended treatment for TD was clonazepam.

## Introduction

Antipsychotic (AP) medication cessation may cause the hyperkinetic movement disorder known as tardive dyskinesia (TD) [1]. A category of complex neurodevelopmental diseases known as autism spectrum disorders (ASD) includes defective or delayed speech, poor social interaction, repetitive, stereotyped behavior/restricted interests, and sensory abnormalities [2]. So, as an approach to the management of irritability associated with ASD, the U.S. Food and Drug Administration (FDA) has approved atypical second-generation antipsychotics, including risperidone and aripiprazole [3,4]. However, a definitive relationship has not been proven; prolonged use of traditional antipsychotics has been linked to tardive dyskinesia [5]. Patients using first-generation antipsychotics (FGAs) have a 4-5% annual incidence of TD [6]. The incidence of TD is thought to range from 2.1 to 4.9% [7] and is related to the use of second-generation antipsychotics (SGAs) [8]. Herein we report a case of a six-year-old male diagnosed with autism and attention deficit hyperactivity disorder (ADHD), who was managed with risperidone; secondary to a sudden stop of risperidone, tardive dyskinesia was induced.

## Case presentation

A six-year-old male patient who had been diagnosed with autism and ADHD at the age of four years presented to King Fahd Central Hospital in Jazan, Saudi Arabia. He started risperidone at the age of four years, his clinical response was good, and he was stable. The patient presented to the emergency department with a history of abnormal movement in the form of unsteady gait, axial dystonia, twisting and spreading of fingers, shoulder shrugging, and protruding tongue associated with hypersalivation. The patient had no fever, eye discharge, double vision, no history of drug ingestion. These symptoms started to appear after two days of a sudden stop of risperidone. The abruption was done by his mother without any instruction from his physician. During admission, the patient was vitally stable. Laboratory investigation tests were performed. Complete blood count (CBC), liver function tests, renal function tests, electrolyte tests, glucose level, and thyroid function tests were done and showed normal levels. In addition, brain magnetic resonance imaging (MRI), computerized tomography (CT), and an electroencephalogram (EEG) showed normal findings. Other causes that induced similar symptoms were ruled out. The diagnosis of tardive dyskinesia was presumed. 

The neurologist did not resume risperidone and started clonazepam with a gradual increase of the dose from 0.2 mg/twice a day for five days to 0.2 mg/three times a day. The patient's symptoms improved within a week; then, he was discharged with a follow-up with a psychiatrist and neurologist.

## Discussion

A novel and unusual antipsychotic drug, risperidone's therapeutic effects are likely due to a dual antagonistic impact on 5-HT2 and D2 dopamine receptors [9,10]. Risperidone has also been demonstrated to have anti-dyskinetic effects [11]. Risperidone can still cause TD in certain patients, although its proportional risk has not yet been determined [12]. Here, we report a new case of tardive dyskinesia induced secondary to a sudden stop of the risperidone medication in n autistic/ADHD child patient.

The TD mechanism has been the subject of numerous theories. Other paths that have been highlighted and are hypothesized to either directly or indirectly alter the nigrostriatal pathway are not limited to dopamine blocking. The striatonigral gamma-aminobutyric acid-ergic (GABAergic) neurons' malfunction, disruption of the dopaminergic and cholinergic systems, hypersensitivity of the dopamine receptor system in the nigrostriatal pathway, and excitotoxicity are highlighted [13].

Clinically, tardive dyskinesia manifests as truncal musculature, limbs, tongue, neck, and facial muscle stereotyped, involuntary movements. Lip-smacking, tongue protrusion, perioral movements, chewing movements, or cheek puffing are examples of buccolingual movements, which include masticatory muscles. Sometimes it may be difficult to tell these motions apart from the stereotypical posturing found in persons with chronic psychosis. However, patients with persistent exposure to dopamine D2 receptor inhibition exhibit tardive dyskinesia [14]. Regarding our patient, he developed a typical manifestation of TD.

Certain laboratory tests and imaging procedures may be used in the evaluation of tardive dyskinesia. In most tardive dyskinesia patients, brain imaging tests like CT and MRI are normal. However, they might help rule out other illnesses, including Fahr syndrome, which causes calcification in the basal ganglia, and Huntington's disease, both of which are characterized by caudate nucleus atrophy [15,16]. Regarding our case, the laboratory and imaging results showed normal findings. Other causes that induced symptoms were ruled out. The diagnosis of TD was presumed. 

The main goal of treating TD is to stop using the substance that caused it whenever possible; however, cautious tapering is advised because abrupt withdrawal increases the risk of developing TD or withdrawal emergent syndrome [17]. This fact has been confirmed in the current case, where the patient's mother suddenly abrupted medication, and after two days, the symptoms of TD developed. 

When possible, dopamine receptor antagonists should be avoided in favor of alternative drugs that are less likely to result in tardive dyskinesia [14]. There are currently not many available treatments. Clonazepam and ginkgo biloba are two of the few treatments that the American Academy of Neurology advises [18]. On April 11, 2017, the FDA approved the use of valbenazine - an inhibitor of vesicular monoamine transport type 2 (VMAT2) to treat tardive dyskinesia [19]. According to the findings of the randomized, double-blind, placebo-controlled phase 3 trial of valbenazine for tardive dyskinesia (KINECT 3 trial), valbenazine significantly reduced tardive dyskinesia when compared to a placebo. However, the best course of action is primary prevention [20]. Regarding our case, risperidone was already stopped by his mother, and clonazepam was started with a gradual increase of the dose from 0.2 mg/twice a day for five days to 0.2 mg/three times a day. Patient symptoms improved within a week, then discharged with follow-up with a psychiatrist and neurologist.

## Conclusions

Our case is a new, uncommon case of tardive dyskinesia induced by the sudden stop of medication during the treatment of an autistic/ADHD patient with risperidone. Risperidone and other atypical second-generation antipsychotics were used to treat autism spectrum disorders. TD is more likely to be triggered by the abrupt discontinuation of risperidone. The chosen laboratory tests and imaging results are helpful in ruling out other causes that induced symptoms and presumed diagnosis of TD. Clonazepam showed a significant impact on the management of TD. Clonazepam and ginkgo biloba are two of the few treatments that the American Academy of Neurology recommends.
